# Cell-SELEX for aptamer discovery and its utilization in constructing electrochemical biosensor for rapid and highly sensitive detection of *Legionella pneumophila* serogroup 1

**DOI:** 10.1038/s41598-024-65075-4

**Published:** 2024-06-19

**Authors:** Aysha Shaukat, Amani Chrouda, Saima Sadaf, Fatimah Alhamlan, Shimaa Eissa, Mohammed Zourob

**Affiliations:** 1 Department of Chemistry, Alfaisal University, 11533 Riyadh, Kingdom of Saudi Arabia; 2 School of Biochemistry and Biotechnology, University of the Punjab, Lahore, Pakistan; 3 King Faisal Specialist Hospital and Research Center, Riyadh, Kingdom of Saudi Arabia; 4 Department of Chemistry, Khalifa University of Science and Technology, 127788, Abu Dhabi, United Arab Emirates; 5 Center for Catalysis and Separations, Khalifa University of Science and Technology, 127788, Abu Dhabi, United Arab Emirates

**Keywords:** Aptamer, SELEX, Biosensors, Legionellosis, Gold electrodes, Square wave voltammetry, Biochemistry, Biological techniques

## Abstract

This study introduces an innovative electrochemical aptasensor designed for the highly sensitive and rapid detection of *Legionella pneumophila* serogroup 1 (*L. pneumophila SG1*), a particularly virulent strain associated with Legionellosis. Employing a rigorous selection process utilizing cell-based systematic evolution of ligands by exponential enrichment (cell-SELEX), we identified new high-affinity aptamers specifically tailored for *L. pneumophila SG1*. The selection process encompassed ten rounds of cell-SELEX cycles with live *L. pneumophila*, including multiple counter-selection steps against the closely related *Legionella* sub-species*.* The dissociation constant (K_d_) of the highest affinity sequence to *L. pneumophila SG1* was measured at 14.2 nM, representing a ten-fold increase in affinity in comparison with the previously reported aptamers. For the development of electrochemical aptasensor, a gold electrode was modified with the selected aptamer through the formation of self-assembled monolayers (SAMs). The newly developed aptasensor exhibited exceptional sensitivity, and specificity in detecting and differentiating various *Legionella* sp., with a detection limit of 5 colony forming units (CFU)/mL and an insignificant/negligible cross-reactivity with closely related sub-species. Furthermore, the aptasensor effectively detected *L. pneumophila SG1* in spiked water samples, demonstrating an appreciable recovery percentage. This study shows the potential of our aptamer-based electrochemical biosensor as a promising approach for detecting *L. pneumophila SG1* in diverse environments.

## Introduction

*Legionella pneumophila* (*L. pneumophila*), a Gram-negative pathogenic bacterium, is prevalent in both natural and artificial hot and humid environments, such as streams, lakes, fountains, cooling towers, hot showers, and plumbing systems. This bacterium serves as a significant causative agent of Pontiac fever and Legionnaires’ disease, leading to respiratory infection and pneumonia, causing multisystem damage in humans^1,2^. As a water-borne pathogen capable of spreading through inhalation of aerosols, it poses substantial threats to immunocompromised individuals, particularly in hospital environments. Its resistance to disinfectants adds complexity to disease control efforts. Persistent efforts are, therefore, being made to develop assays for rapid and accurate detection of *L. pneumophila* in various environments, in particular hospitals/tertiary healthcare facilities and water reservoirs, for effective disease surveillance and to reduce fatalities.

Conventional methods employed for *Legionella* detection, including culture-based techniques, enzyme-linked immunosorbent assays (ELISA), micro-agglutination (MA) and indirect fluorescent antibody (IFA)-based assays, are hindered by limitations in sensitivity, specificity, and/or turnaround time^3,4^. Polymerase chain reaction (PCR), especially on-site PCR, offers a more straightforward and reliable method for *Legionella* identification in field settings. However, its accuracy is notably higher for *Legionella pneumophila* compared to non-*pneumophila* sub-species^5^. Unfortunately, PCR techniques are not suitable for field applications and can be time-intensive. The direct fluorescent antibody (DFA) assay represents a rapid test with a sensitivity of 71% for *L. pneumophila* serogroup 1 (SG-1) and a specificity approaching 99%. Nevertheless, this method demands extensive expertise and labor to execute^6,7^. Another sensitive approach for *Legionella* detection is loop-mediated isothermal amplification (LAMP) based on electrochemical (EC) transduction principles^8^. LAMP is faster and less labor-intensive compared to traditional methods^8–11^. However, it involves the use of several costly reagents such as primers and enzymes.

Biosensors, in particular electrochemical biosensors, have emerged as a promising alternative to conventional techniques for bacterial detection owing to the sensitivity afforded by state-of-the-art electrochemical techniques. Their appeal lies in their ability to rapidly analyze samples, and discriminate analytes within complex biological/environmental matrices with exceptional specificity and sensitivity, all achieved at a relatively cost-effective rate compared to the conventional methods. For instance, Park et al. elucidated the utilization of zinc oxide nanorods on a Ti/Au electrode for highly sensitive *L. pneumophila* detection with specific antibodies^12^. Additionally, Li et al. introduced a dual electrochemical and fluorescent immunosensor platform tailored for the selective detection of *L. pneumophila*. This platform involved the covalent immobilization of fluorophore-conjugated antibodies onto gold (Au) chips^13^. However, the use of antibodies in these immunosensors poses limitations on their stability, batch-to-batch variations and complex in vivo production, thereby restricting their practical applicability.

In recent years, aptasensors that utilize aptamers as recognition elements, have garnered significant attention among researchers particularly of those involved in diagnostics. Aptamers, small single-stranded DNA or RNA molecules, exhibit high specificity and affinity in binding to target molecules. They have facilitated the detection of various pathogens such as *Escherichia coli, Salmonella, Listeria monocytogenes*, *Campylobacter jejuni,* severe acute respiratory syndrome coronavirus 2, influenza virus, and human immunodeficiency virus^14–20^, from a variety of samples.

Despite considerable research on biosensors for detecting *L. pneumophila*, only a few recent cases of aptamer selection have been reported^21^. These aptamers were subsequently utilized to develop a surface plasmon resonance (SPRi) aptamer-based titration assay^22^. The SPR assay necessitates expensive instrumentation, thereby escalating the cost of the assay and posing challenges for its use on a large scale.

In the present research, we report the selection of new aptamer against *L. pneumophila SG-1* using cell SELEX (systematic evolution of ligands by exponential enrichment) with higher affinity. Negative selection was conducted utilizing close-related subspecies to ensure target-specific aptamers selection. By combining the properties of DNA aptamers with the high sensitivity of electrochemical techniques, we have developed an electrochemical aptasensor platform for the label-free, sensitive, specific, and rapid detection of *L. pneumophila SG-1*. This represents the first electrochemical aptasensor for *L. pneumophila SG-1*, holding the promise of enabling early, specific, and cost-effective detection of *L. pneumophila SG-1* in various clinical and environmental settings. This advancement could significantly enhance surveillance and aid in the effective control of infectious outbreaks.

## Materials and methods

### Chemicals and reagents

The chemicals used in this work are described in details in the supporting information file. The 45 µm Spin-X centrifuge filter tubes were purchased from Corning Life Sciences (MA, USA), whereas, 0.5 mL Amicon ultra-centrifugal filter were acquired from EMD Millipore. Random DNA library and the labeled and unlabeled oligos were purchased from Metabion International (Planegg, Germany). The binding buffer was composed of 50 mM Tris (pH 7.5), 2 mM MgCl_2_, and 150 mM NaCl prepared in deionized water. A solution of 7 M urea prepared in the binding buffer was utilized as elution buffer to liberate the sequence bound to the target. Tris–EDTA (TE) buffer was composed of 10 mM Tris (pH 7.4) and 1 mM EDTA prepared in deionized water.

### Instrumentation

The T100 Thermal Cycler (Bio-Rad, USA) was used for amplification reactions. The DNA concentration was measured using Thermo Scientific™ NanoDrop™ 2000C spectrophotometer while NanoDrop™ 3300 was used to get the fluorescence data. The AUTOLAB PGSTST302N potentiostat/galvanostat (Metrohm, Netherlands) with an impedance module was utilized to conduct cyclic voltammetry (CV) and electrochemical impedance spectroscopy (EIS) investigations. The NOVA 1.11 program was employed for both data collection and analysis. Impedance data were collected at a direct current (dc) voltage of 10 mV amplitude, with amplitude and phase angle of the resulting current, recorded across 100 kHz to 0.1 Hz frequencies. The CV was scanned from −0.2 to + 0.6 V at a rate of 0.1 V/s, conducted within a redox solution containing 5 mM K_3_[Fe(CN)_6_]/K4[Fe(CN)_6_] in 1:1 ratio and 0.1 M KCl as a supporting electrolyte. Square wave voltammetry (SWV) observations were recorded using an amplitude of −20 mV, an interval length of 0.04 s, a step potential of 5 mV, a scan rate of 125 mV/s, and a frequency of 25 Hz. The screen-printed gold electrodes (SPE-Au) were sourced from BioDevice Technology (Ishikawa, Japan). DEP-Chip consists of a polymeric substrate on which a gold working electrode, a platinum counter electrode, and a silver/silver chloride as reference electrode.

### Bacterial strains and culture conditions

The TOPO TA cloning kit along with ultracompetent *E. coli* cells was purchased from Invitrogen Inc. (NY, USA). The *Legionella* bacteria, i.e., *L. pneumophila* (ATCC 33152), *L. anisa* (ATCC 325292), *L. micdadei* (ATCC 33218), *L. bozemanae* (ATCC 33217), and *L. dumoffii* (ATCC 33279) were acquired from the American Type Culture Collection Centre (VA, USA). To achieve a quantifiable count of 5 × 10^4^ CFU/mL in each instance, the sub-species (obtained in lyophilized form) were cultivated on agar plates containing buffered charcoal yeast extract (BCYE), incubated at 37° C incubator with 5% CO_2_ and 60% humidity for 24–72 h. Each Legionella species’ culture stock was either immediately used for further analysis or kept in an ultralow temperature freezer (−80° C) until needed.

### DNA library preparation and PCR amplification

A DNA library was constructed with a 40-nucleotide random region and 16-nucleotide flanking sequences at both 5′ and 3′ ends (5′-TCCCTACGGCGCTAAC-N40-CCACCGTGCTACAAC-3′) to serve as primers for PCR amplification and facilitate the selection process based on cell-SELEX. During the selection process, the fluorescein-labeled forward primer (16-mer) measured and amplified the DNA, while the reverse primer (16-mer) linked to a hexamethylene glycol spacer and 20 poly-A oligonucleotides blocked extension during polymerase chain reaction (5′-polydA20-HEG-GTTGTAGCACGGTGGC-3′). We utilized symmetric and asymmetric polymerase chain reactions. The process started with symmetric PCR and then moved onto asymmetric PCR using the same product. Each of the two PCR tubes was filled to a total of 100 μL with deionized water, and 50 μL of master mix, which included 10X buffer (10 μL), MgCl_2_ (6 μL), dNTPs (2 μL), Taq polymerase (1 μL), DNA sample (15 μL), forward primer (2.5 μL), and reverse primer (2.5 μL), was prepared for the symmetric PCR. Following are the parameters used for symmetric and asymmetric polymerase chain reactions: In Step 1, the temperature is set to 95 °C for 5 min. In Step 2, the temperature is varied between 47 °C for 1 min and 72 °C for 1 min. This is followed by 15 cycles of these temperatures, ending with a final extension step at 72 °C for 10 min. For the asymmetric PCR, ten PCR tubes were made, with fifty microliters of mixture components in each. Up to the tenth cycle of SELEX, the amplicon from the asymmetric PCR was concentrated, purified, and stored. After that, the eluted ssDNA was amplified using unlabeled primers. Afterwards, in the next step cloning was introduced with the resulting product of this stage.

### Whole cell-SELEX for *L. pneumophila SG-1* aptamer selection

In order to selectively target live bacterial cells and select aptamers developed for *L. pneumophila SG-1*, a tailored strategy of whole cell-SELEX was used. The procedure started with three or four washes with deionized water on the pristinely collected bacterial colonies. Afterwards, a pretreated DNA library solution was poured onto the rinsed bacterial cells. To carry out the pretreatment stage, 150 pmol of DNA solution was heated in 300 µl of binding buffer at 90 ℃ (5 min), cooled to 4℃ (10 min), and then allowed to stand at room temperature (5 min). Thereafter, mixture was incubated for 2 h while being gently turned over end-to-end rotation. Five washes with binding buffer were used to remove any unbound DNA from the mixture. Then, elution buffer (300 µl), heated at 90 °C for 5 min, was applied to elute the bound DNA. This step was repeated until no fluorescence was detected in the washes. The eluted DNA was then desalted and PCR ampilified to be utilized in the subsequent cycle. After the fifth cycle of the cell-SELEX procedure, several counter-selection stages were added to improve specificity. As mentioned above, a total of five counter-selections were conducted utilizing sub-species of *Legionella* that are closely related.

To clone the enriched single-stranded aptamer pool into *E. coli* DH5α T1R cells, the TOPO DNA cloning kit was employed following the manufacturer's instructions; the procedure was stopped after the tenth cycle. The pool was desalted and amplified using unlabeled primers. To amplify the cloned DNA inserts, the blue-white colonies were selected and amplified by colony PCR using M13 universal primers. After running the PCR on a 2% agarose gel, the positive transformants containing the target sequence were further examined using Sanger's dideoxy sequencing method. The selected sequences for *L. pneumophila SG-1* were aligned using a web-based software (http://www.ibi.vu.nl/programs/praline).

### Calculating equilibrium dissociation constants (K_d_) for *L. pneumophila SG-1 aptamers*

By incubating FITC-labeled aptamers at various concentrations (0–300 nM) in the presence of a constant cell concentration of *L. pneumophila SG-1* (5 × 10^4^ CFU/mL), the binding affinity of the aptamers to *L. pneumophila SG-1* was determined. The freshly collected bacteria were combined with aptamers and left to incubate at 37 ℃ for one hour. To remove the unbound DNA, the cells were washed with the binding buffer (Supplementary Material) three times. To elute the cell-bound DNA, 300 µl of elution buffer was added and heated to 80 ℃ for 5 min. After centrifugation, the fluorescence intensity of the elution was measured using FluorDrop-3300. Using the GraphPad Prism, the equilibrium dissociation constants (K_d_) of the aptamers was calculated by non-linear regression analysis.

### Secondary structure prediction

The chosen aptamers’ secondary structures were anticipated with the help of the MFOLD program. A temperature of 25 ℃ and a buffer composition of [Na^+^] = 150 mM and [Mg^++^] = 2 mM were utilized for the secondary structure prediction. The program determined the aptamer stability and folding patterns by computing the free energy (ΔG) of the chosen ssDNA.

### Preparation of aptasensors for *L. pneumophila* SG-1

10 µL of 1 mM of the thiolated aptamer solution was incubated on the gold electrode overnight. To remove the aptamers that had not been adsorbed, the electrodes were washed with a 0.1 M Tris-HCl buffer (pH 7.4). Subsequently, the electrodes surfaces were incubated for 30 min at room temperature in a 1-Mercapto-1-hexanol (6-MCH) solution prepared in PBS buffer. This step aimed to optimize the distribution and orientation of the aptamer onto the electrode surface, minimize nonspecific binding, and block unreactive surfaces. Following the formation of a self-assembled monolayer (SAM), the electrodes were rinsed several times with a Tris-HCl buffer solution and then dried using a stream of pure nitrogen.

### Detection of *L. pneumophila* SG-1 and data analysis

The aptasensors were tested against *L. pneumophila SG-1* in PBS buffer at different concentrations. After incubating the electrodes for 30 min with serial dilutions of the bacterial strains (in CFU/mL), the electrochemical signals were measured. We conducted all our experiments under optimized conditions.

### Utilization of the *L. pneumophila* SG-1 aptasensor on real samples

The aptasensor was tested in samples that were purposely contaminated with known concentrations of *Legionella pneumophila* SG-1*,* ranging from 10^1^ to 10^7^ CFU/mL to evaluate its performance in real-world analytical applications. The spiked samples were introduced to the aptasensor, and SWV was utilized to record the electrochemical signal.

## Results and discussion

### Whole cell-SELEX for specific aptamers selection against *L. pneumophila* SG-1

**Figure 1:**
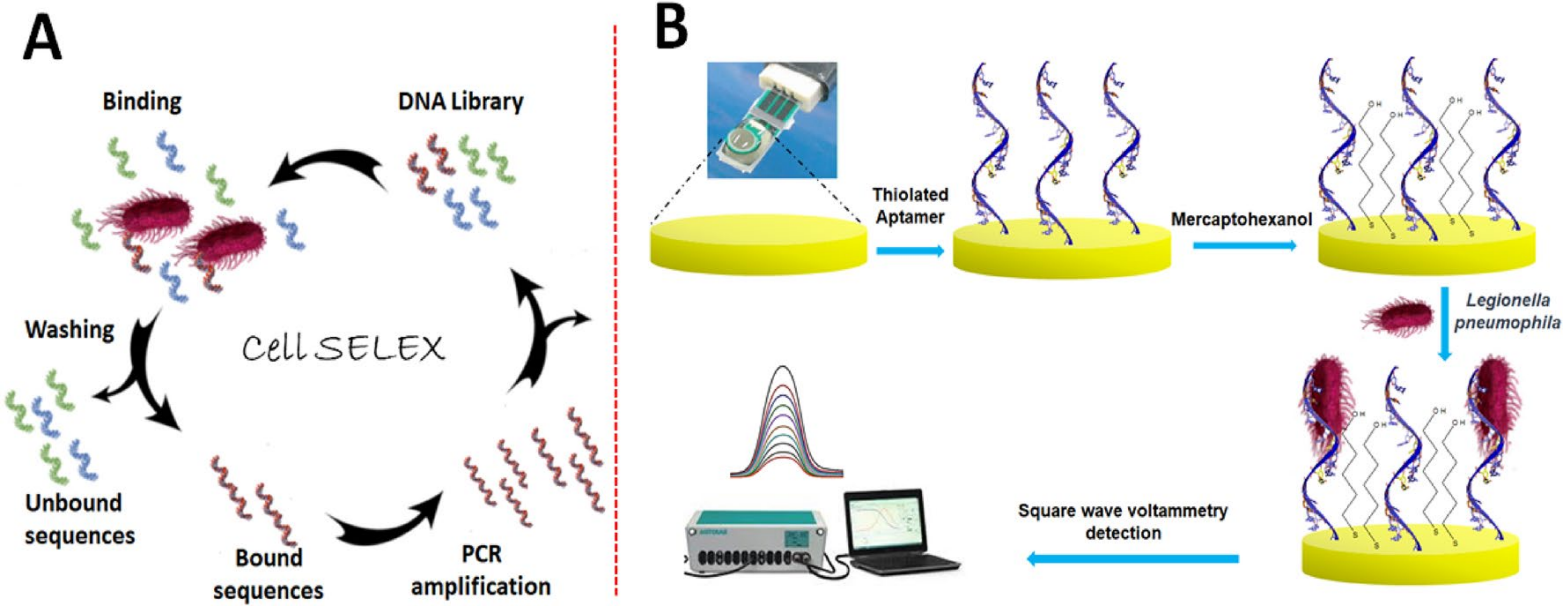
(**A**) Schematic of the cell SELEX protocol for the aptamer selection, (**B**) the fabrication process of the aptasensor.

**Figure 2:**
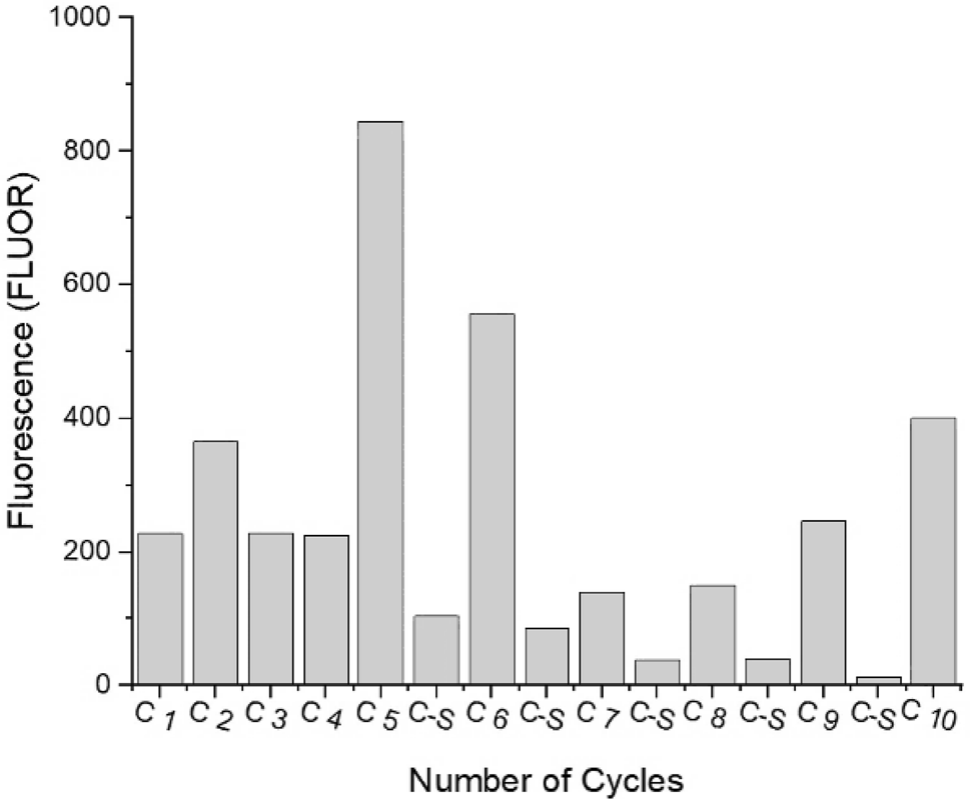
Recovery of the DNA during cell-SELEX process resulting in the enrichment of aptamers specific for *L. pneumophila SG-1*. The data presented in bar-graph shows the fluorescence intensity values of the ssDNA, eluted in each cycle. Counter-selection (C-S) was introduced after fifth cycle to onwards to remove non-specifically bound DNA.

In this study, a whole cell-SELEX based approach was used to produce aptamers specifically targeting *L. pneumophila SG-1* (Fig. 1A). During each selection round, single-stranded DNA library was incubated with the *L. pneumophila SG-1* bacterial cells, followed by washing to remove unbound DNA and elution to recover the bounded DNA. Subsequently, the bounded DNA was PCR amplified and purified to generate a new pool, which was then utilized for subsequent cycles of selection. Notably, a distinctive counter-selection strategy was also employed to ensure generating *L. pneumophila SG-1*-specific DNA aptamers. The counter-selection process was carried out against closely related subspecies. The bar graph illustrated in Fig. 2 displays a steady increase in the concentration of DNA recovered for *L. pneumophila,* during each progressive cycle of cell-SELEX.

Noteworthy, non-specific DNA sequences were effectively removed through counter-selection steps after the 5, 6, 7, 8, and 9th cycles of Cell-SELEX. The previous aptamer selection for *L. pneumophila*, incorporated a counter-selection step involving only two Pseudomonas species^21^. Our more stringent conditions by performing counter selection against the most related *Legionella* species (*L. pneumophila SG-3, L. anisa, L. micdadei, F. bozemanae, and F. dumoffii)* are intended to augment the specificity of the new aptamer. The Cell-SELEX procedure was concluded until reaching a plateau and the DNA eluted from the 10th round was utilized for cloning, followed by sequencing.

### Aptamers characterization by assessing the relative binding affinity to *L. pneumophila* SG-1

**Figure 3:**
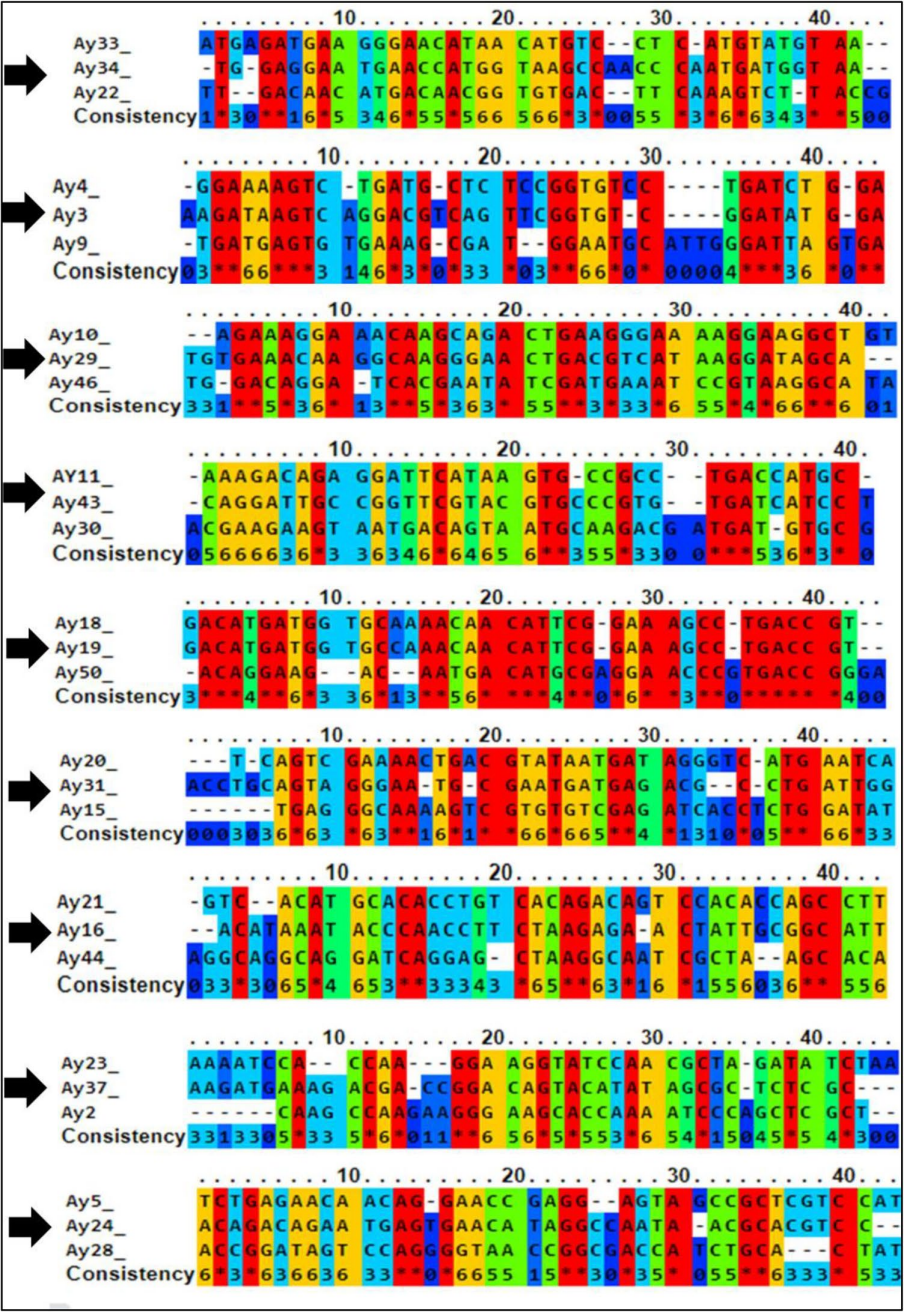
Multiple sequence alignment of sequences selected for *L. pneumophila SG1* by using a random 40 neoclotide DNA library. Conserved regions among multiple sequences are highlighted in red. Arrow shows the sequence selected for further binding affinity studies.

The selected sequences (40 nucleotides after omitting the primers binding sites) were aligned using a multiple sequence alignment software, PRALINE. Subsequently, the sequences were organized (grouped) into sub-groups, each containing three sequences. Specific sequences targeting *L. pneumophila SG1* were selected based on conserved homology. These sequences include AY-34, AY-3, AY-29, AY-43, AY-19, AY-31, AY-16, AY-37, and AY-24 (Fig. 3).

**Figure 4:**
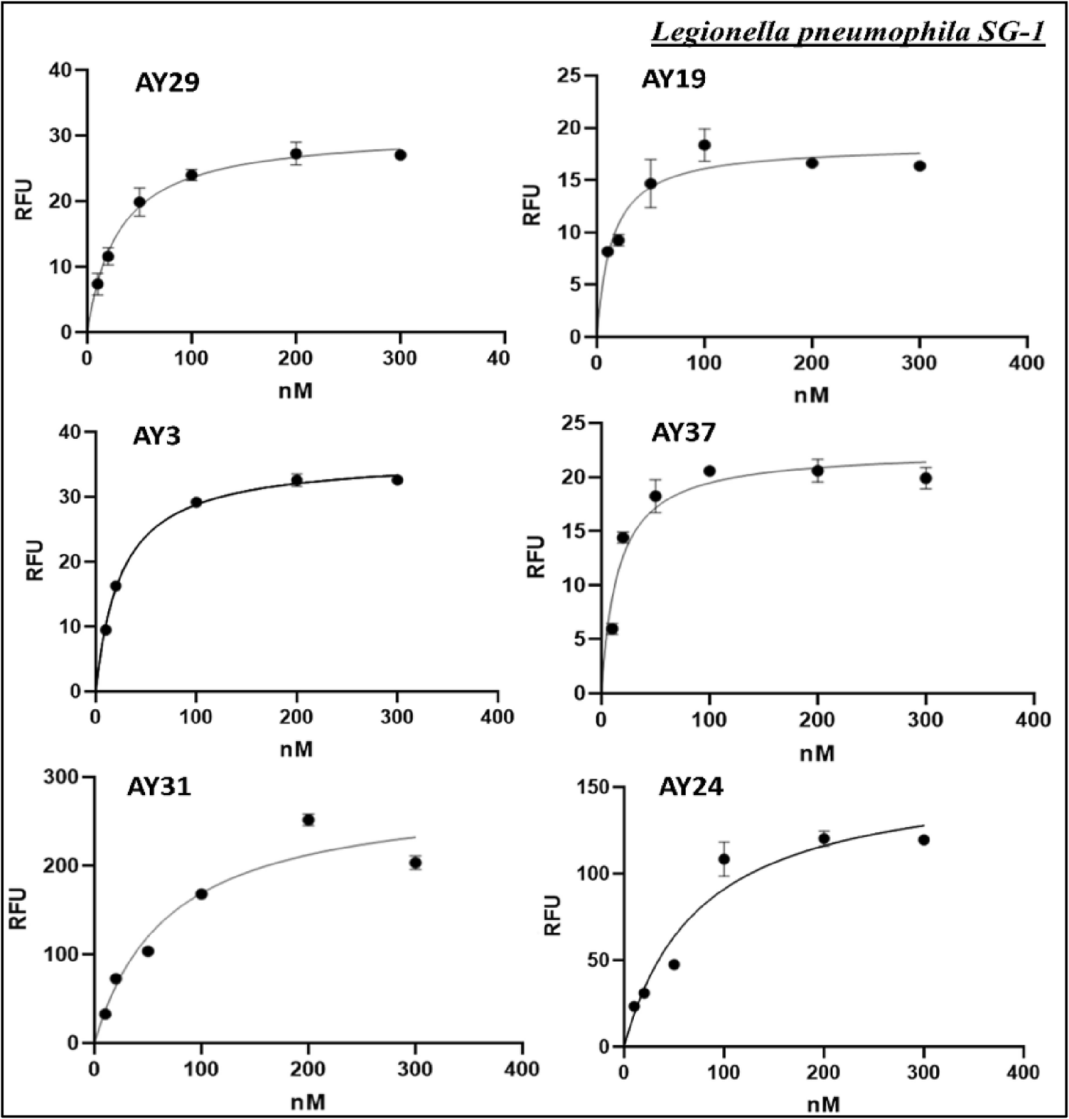
The binding curves (a plot of the fluorecence intensity versus aptamer concentration) used to calculate the K_d_ for *L. pneumophila SG-1* selected apatmers. The K_d_ of selected aptamers was measured on the basis of non-linear regression analysis. AY3: K_d_ = 15.61 nM; AY37: K_d_ = 17.96 nM; AY31: K_d_ = 68.83 nM; AY29: K_d_ = 17.38 nM; AY24: K_d_ = 75.24 nM; and AY19: K_d_ = 14.19 nM are shown. AY19 was selected for further analysis on the basis of its least K_d_ value.

From the pool of identified sequences, nine aptamers were selected for in-depth analysis. The relative binding affinity was measured using a 5′-end FITC labeled aptamers. The K_d_ value of the selected aptamers was determined through a fluorescence binding-assay. Different concentrations of aptamers were allowed to interact with a constant number of bacteria and the resulting fluorescence was measured after removing the unbound aptamer and eluting the bound DNA. Figure 4, presents K_d_ values of six *L. pneumophila*-specific aptamers that are ranging between 14 and 75 nM with the least K_d_ exhibited by AY-19 (14.19 nM). However, aptamers AY-34, AY-43 and AY-16 have shown higher K_d_ values in the micromolar range indicating lower affinity to *L. pneumophila.*

It is pertinent to note that the K_d_ values for the aptamers selected in this study, is significantly lower than the aptamers described by Saad et al.^21^. The reported aptamer has shown a K_d_ of 116 nM which is around ten-times higher than our AY19 aptamer (K_d_ = 14.19 nM).

**Figure 5:**
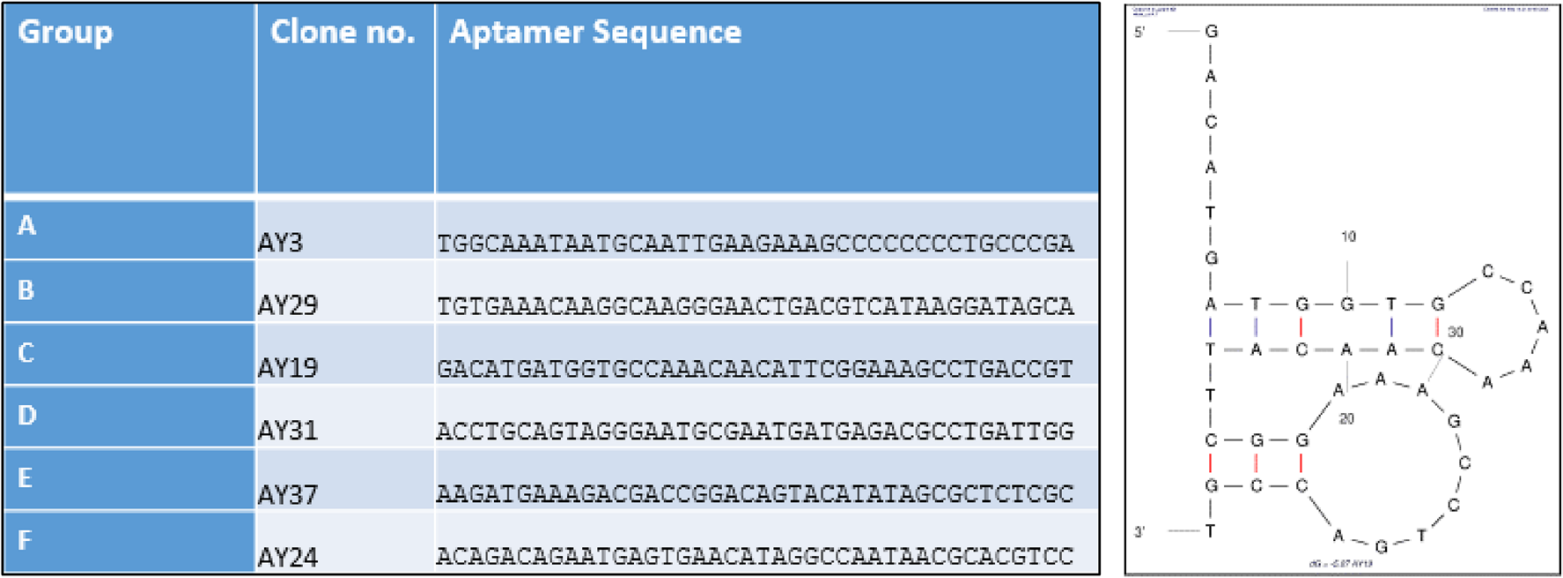
Sequences of aptamers selected for *L. pneumophila* SG-1 (left panel). The secondary structure of *L. pneumophila SG-1* aptamer AY-19 is shown (right panel). AY-19 structure has stems and loops. The mfold software was utilized for the prediction of secondary structure.

Based on the superior binding affinity for *L. pneumophila SG-1*, the aptamer AY19 was chosen among the other six sequences shown in Fig. 5 for further comprehensive analysis. The predicted secondary structure of the AY19 aptamer contains a stem-loop motif (Fig. 5, right panel), with least Gibb’s free energy (ΔG).

### Fabrication of the electrochemical aptasensor

Following the SELEX process, aptamer AY-19, exhibiting the lowest K_d_, was selected for the fabrication of a label-free electrochemical aptasensor. The fabrication process, illustrated in Fig. 1, involved the immobilization of thiolated aptamer onto a gold screen-printed electrode through self-assembly (step 2), followed by the blocking of free gold sites with MCH (step 3). Detection relied on the specific binding of *L. pneumophila SG-1* to the aptamer immobilized on the electrode surface, resulting in the hindrance of charge transfer of the redox probe, which was monitored via SWV (step 4). The aptasensor’s response to various concentrations of the bacteria was studied, with changes in the SWV peak current serving as the basis for detection.

### Characterization of aptasensor construction

**Figure 6:**
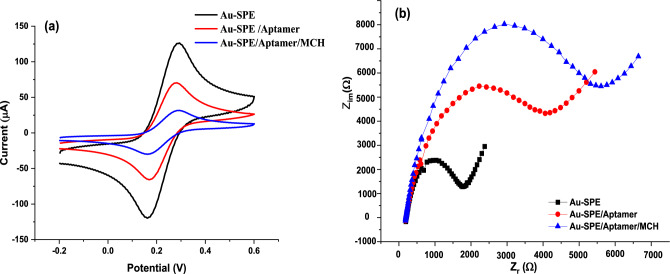
Cyclic voltammetry (**a**) and the EIS-based Nyquist plot (**b**) of different surface modifications.

Electrochemical measurements such as cyclic voltammetry and electrochemical impedance spectroscopy (EIS) were employed to monitor the various modifications steps. These measurements were performed using a ferro/ferricyanide redox probe, and the results are presented in Fig. 6. The figure illustrates that the bare electrode displays a distinct reversible redox peak (black line) in CV, indicating the cleanliness of the surface. Upon surface modification with aptamer AY-19, the redox peaks decrease (red curve). This reduction can be ascribed to the formation of an organized structure between aptamers, combined with the negative charge of the aptamer’s phosphate backbone, acting as a barrier hindering electron transfer^23–25^. The mercaptohexanol (MCH) self-assembled monolayer induces a further reduction in the current (blue line) by blocking the available gold sites. Additionally, it has been documented that MCH enhances the alignment of the thiolated aptamer.

The EIS is recognized as the predominant technique for characterizing electrodes, providing valuable insights into the chemical modifications applied to the Au-SPE surface, thereby altering its electrical properties. EIS measurements allowed us to comprehend various aspects, including the charge transfer from a solution to the electrode surface, solution resistance, diffusional transport of species to and from the bulk solution, and the formation of double-layer capacitance.

The Nyquist plot (Fig. 6b) featured a semicircle part, corresponding to the redox probe electron charge-transfer resistance (R_ct_). This R_ct_ exhibited an inverse relationship with the rate of electron transfer^26^. Additionally, a linear part, known as Warburg impedance, representing a diffusion-limited process, was observed^27^.

The bare Au electrode exhibited a small semicircle domain, indicating fast electron transfer and a primarily mass diffusion-limited step. Upon immobilization of the aptamer AY-19 probe on the bare Au SPE surface, the R_ct_ increased significantly. This increase in R_ct_ could be attributed to electrostatic repulsion between the negatively charged aptamer and a negatively charged redox couple, thereby minimizing the electrode transfer event. Subsequent surface modification with MCH further expanded the diameter of the semicircle in the Nyquist plot, indicating the blocking of free gold sites on the electrode surface. Thus, both the EIS and CV data mutually supported and validated each other, confirming the presence of chemical structure alteration at the gold (Au) surface throughout at every step of the aptasensor design.

### Electrochemical detection of *L. pneumophila* SG-1

**Figure 7:**
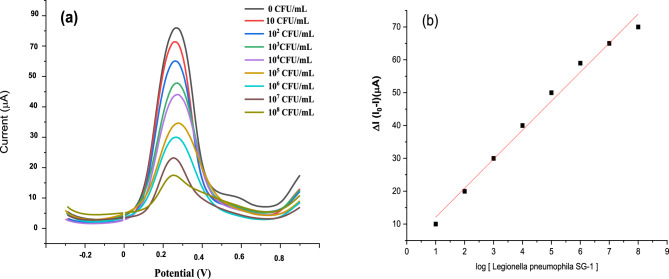
(**a**) Square wave voltammetry (SWV) measurements of the AY19 aptasensor variation versus cumulative *L. pneumophila* SG-1 cells concentration; and (**b**) the corresponding calibration curves.

The performance characteristics of the aptasensors were assessed through the generation of calibration curves derived from SWV measurements, as illustrated in Fig. 7. The experiments were carried out under optimal conditions selected for their effectiveness. The SWV was a chosen analytical technique due to its inherent advantages, including heightened sensitivity, rapid analysis, and significant reduction in capacitance currents^28,29^.

The prepared aptasensor was exposed to varying concentrations of *L. pneumophila SG-1* for a duration of 30 min. The resulting peak current intensities (I), obtained from the signals of the redox probe, were then correlated with the concentrations of *L. pneumophila SG-1*; higher concentrations *L. pneumophila* corresponded to a decrease in peak currents (Fig. 7a). This phenomenon was ascribed to the rising charge transfer resistance associated with the increasing *L. pneumophila SG-1* concentration in each standard solution. The output electrochemical signal, correlating the aptasensor response (∆I) with the logarithm of *L. pneumophila SG-1* cell concentrations, were generated (Fig. 7b). The aptasensor response was calculated as the variance in the peak current values (∆I = i_o_−i), where i_o_ is the peak current of the background signal of the aptasensor and i shows the current after incubation with varying concentrations of the bacterial cells.

The calibration curve, represents a linear trend ranging from 10 to 10^8^ CFU mL^−1^ was established (Fig. 7a). The aptasensor limit of detection (LOD) was estimated in the order of 4.6 CFU/mL. To assess the reproducibility of the sensors, triplicate measurements were performed, and the experimental results have demonstrated a relative standard deviation of 3.7%. These results unequivocally demonstrate the exceptional sensitivity, repeatability, and stability of the proposed aptasensor.

### Cross-reactivity study of the *L. pneumophila* SG-1 aptasensor

**Figure 8:**
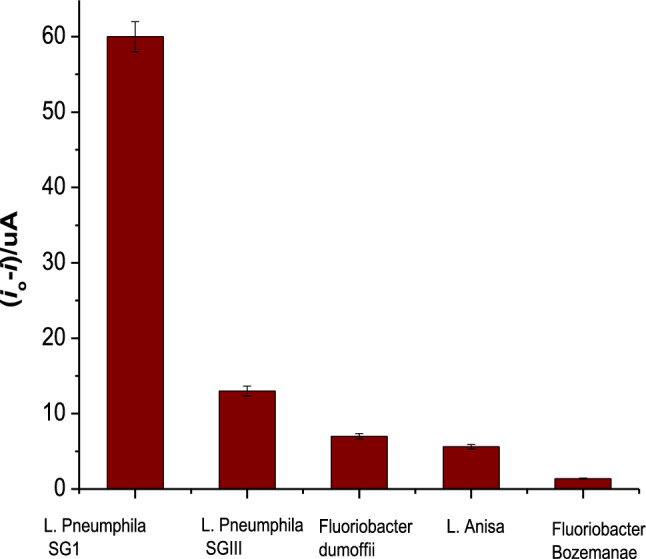
Selectivity investigation of aptasensor by employing various *Legionella Sp.,* like *L. pneumophila* SG3, *L. anisa, F. dumoffii,* and *F. bozemanae.*

To ascertain the selectivity and reliability of the aptasensor towards *L. pneumophila* SG-1, cross-reactivity experiments were conducted against various pathogenic cultures, including *L. pneumophila* SG-3, *L. anisa, F. dumoffii*, and *F. bozemanae*, all maintained at a concentration of 10^6^ CFU/mL. The results of these experiments, illustrated in Fig. 8, demonstrated the aptasensor’s robust selectivity against the target pathogen. Notably, the presence of other bacteria, alongside *L. pneumophila SG-1*, at equal concentrations had no discernible impact on the outcomes when compared to assays conducted solely in the presence of *L. pneumophila SG-1*. This data features the aptasensor’s specificity, affirming its ability to effectively discriminate and identify *L. pneumophila SG-1* even in the presence of other pathogenic cultures, thereby validating its potential for accurate and reliable pathogen detection.

### Evaluation of *Legionella* pneumophila SG-1 aptasensor performance in real samples

To assess the practical utility of the developed aptasensor, it was applied to water samples spiked with known concentrations of bacteria. The aptasensor was then incubated with the spiked samples, and the resulting electrochemical responses were recorded using SWV. The water samples were spiked with varying concentrations of *L. pneumophila SG-1* (10^1^, 10^3^, and 10^7^ CFU/ mL). The obtained results are shown in Table 1, indicated recoveries ranging from 103% to 87.5%, with associated relative standard deviations (RSD, n = 3) in the range of 4.3 to 7.8%. These findings demonstrate the accuracy and reliability of the aptasensor, essential factors to consider for potential real-world applications.

**Table 1:** The recovery percentages obtained using the aptasensor in spiked water samples.

Samples	Concentration (CFU/mL)	Recovery rate (%)	Relative standard deviation (%)
Water	10^1^	103	4.3
	10^3^	91.4	5.4
	10^7^	87.5	7.8

## Conclusions

This study employed whole-cell SELEX methods to isolate and characterize new single-stranded DNA aptamers with high affinity and specificity targeting *Legionella pneumophila SG1*. Counter selections against closely related *Legionella* subspecies contributed to the aptamer's high selectivity. The resulting aptamer exhibited a dissociation constant of 14.2 nM, which is 10 times lower than the reported aptamers, demonstrating remarkable affinity. These aptamers were then incorporated into the design of a novel electrochemical aptasensor, which exhibited an impressive lower detection limit of 5 CFU/ml. These findings highlight the excellent sensitivity of the developed biosensor. The results unequivocally suggest that the selected aptamer holds significant potential as a reliable and user-friendly tool for identifying and detecting *L. pneumophila SG1*. The successful application of this aptamer paves the way for innovative medical devices and assays, streamlining and expediting infection diagnosis in healthcare settings and the environment. This advancement represents a significant step toward more efficient and dependable diagnostic solutions.

### Supplementary Information

Supplementary Information.

## Data Availability

The data that support the findings of this study are available from the corresponding author upon reasonable request.
