# Author Correction: Cell-SELEX for aptamer discovery and its utilization in constructing electrochemical biosensor for rapid and highly sensitive detection of *Legionella pneumophila* serogroup 1

**DOI:** 10.1038/s41598-024-66993-z

**Published:** 2024-07-10

**Authors:** Aysha Shaukat, Amani Chrouda, Saima Sadaf, Fatimah Alhamlan, Shimaa Eissa, Mohammed Zourob

**Affiliations:** 1 Department of Chemistry, Alfaisal University, 11533 Riyadh, Kingdom of Saudi Arabia; 2 School of Biochemistry and Biotechnology, University of the Punjab, Lahore, Pakistan; 3 King Faisal Specialist Hospital and Research Center, Riyadh, Kingdom of Saudi Arabia; 4 Department of Chemistry, Khalifa University of Science and Technology, 127788 Abu Dhabi, United Arab Emirates; 5 Center for Catalysis and Separations, Khalifa University of Science and Technology, 127788 Abu Dhabi, United Arab Emirates

Correction to: *Scientific Reports* 10.1038/s41598-024-65075-4, published online 19 June 2024

The original version of this Article contained an error in the name of Amani Chrouda, which was incorrectly given as Amani Chroudah.

The original Article has been corrected.

