# Supplementary material for: Cell-SELEX for aptamer discovery and its utilization in constructing electrochemical biosensor for rapid and highly sensitive detection of Legionella pneumophila serogroup 1
Source: Sci Rep. 2024 Jun 19;14:14132. doi: 10.1038/s41598-024-65075-4 (PMC11187191; doi:10.1038/s41598-024-65075-4)
Supplement: Supplementary file 1 — Supplementary Information. [file 41598_2024_65075_MOESM1_ESM.docx]

**Supporting Information**

**Cell-SELEX for Aptamer Discovery and its Utilization in Constructing Electrochemical Biosensor for Rapid and Highly Sensitive Detection of *Legionella pneumophila Serogroup 1***

Aysha Shaukat^1^, Amani Chroudah^3^, Saima Sadaf^2^, Fatimah Alhamlan^4^, Shimaa Eissa^5,6^, Mohammed Zourob^1,^*

^1^Department of Chemistry, Alfaisal University, Riyadh 11533, KSA.

^2^ School of Biochemistry and Biotechnology, University of the Punjab, Lahore, Pakistan

^3^ Department of chemistry, College of Science at Zulfi, Majmaah University, 11952, Saudi Arabia

^4^ King Faisal Specialist Hospital and Research center, Riyadh, KSA.

^5^Department of Chemistry, Khalifa University of Science and Technology, Abu Dhabi, P.O. Box 127788, United Arab Emirates

^6^ Center for Catalysis and Separations, Khalifa University of Science and Technology, Abu Dhabi P.O. Box 127788, United Arab Emirates

*Corresponding author:

mzourob@alfaisal.edu

- 1. **Materials and reagents**

Sodium chloride (NaCl), magnesium chloride (MgCl_2_), phosphate-buffered saline pH 7.4 (PBS), tris(hydroxymethyl)aminomethane (tris-base), boric acid, ethylenediaminetetraacetic acid (EDTA) disodium dehydrate, acrylamide/bisacrylamide (30% solution), tetramethylethylenediamine (TEMED), urea, ammonium persulfate (APS), sodium acetate, sodium bicarbonate, sodium azide, hydrochloric acid, bovine serum albumin (BSA), sodium carbonate anhydrous, sodium bicarbonate, dipotassium hydrogen orthophosphate, potassium dihydrogen orthophosphate, ethanol and N, N-dimethyl formamide (DMF) were all procured from Sigma-Aldrich (St Louis, MO, USA). Taq buffer, dNTPs, Taq plus polymerase for PCR amplification, and 100-base pair ladder were purchased from ACE Biotech (Riyadh, Saudi Arabia). Bromo-4-chloro(3-indolyl-b-D-galactopyranoside, X-Gal was obtained from Bio Basic Inc. (Toronto, ON, Canada). Ampicillin lyophilized powder was acquired from Bio-Rad (Hercules California, USA). The bacterial culture plates were supplied by Saudi Prepared Media Laboratory Company (Riyadh, Saudi Arabia). For agarose gel electrophoresis, 50X TAE buffer and agarose powder were sourced from Bio-Rad (Hercules, California, United States). Spin-X cellulose acetate centrifuge filter tubes with a pore size of 45 µm were sourced from Corning life sciences (Tewksbury, MA USA). Amicon Ultra Centrifugal Filter (0.5 mL) was obtained from EMD Millipore (Sigma MA USA; https://www.merckmillipore.com). HPLC purified labeled, and unlabeled oligonucleotides and random DNA libraries were purchased from Metabion International (Planegg, Germany; http://www.metabion.com). E. coli competent cells and the TOPO TA cloning kit containing the pCR2.1-TOPO vector were acquired from Invitrogen Inc. (New York, NY, USA). For the washing step during aptamer selection and detection experiments, the binding buffer consisted of 2 mM MgCl_2_, 50 mM Tris (pH 7.5), and 150 mM NaCl in distilled water. Elution buffer, used to release aptamer sequences bound to the target, consisted of 7M urea in binding buffer. Tris-EDTA (TE) buffer used to elute ssDNA from the denaturing gel was prepared by mixing 1 mM EDTA and 10 mM Tris (pH 7.4) in distilled water.
